# Intrauterine Exposure to Cadmium Reduces HIF-1 DNA-Binding Ability in Rat Fetal Kidneys

**DOI:** 10.3390/toxics6030053

**Published:** 2018-09-03

**Authors:** Tania Jacobo-Estrada, Mariana Cardenas-Gonzalez, Mitzi Paola Santoyo-Sánchez, Frank Thevenod, Olivier Barbier

**Affiliations:** 1 Departamento de Toxicología, Centro de Investigación y de Estudios Avanzados del Instituto Politécnico Nacional, 07360 Ciudad de México, Mexico; tjacoboe@ipn.mx (T.J.-E.); santoyomitzi@gmail.com (M.P.S.-S.); 2 Departamento de Sociedad y Política Ambiental, CIIEMAD, Instituto Politécnico Nacional, 07340 Ciudad de México, Mexico; 3 Renal Division, Department of Medicine, Brigham and Women’s Hospital, Harvard Medical School, Boston, MA 02115, USA; mcardenasgonzalez@bwh.harvard.edu; 4 Department of Physiology, Pathophysiology and Toxicology and ZBAF (Center for Biomedical Education and Research), Faculty of Health-School of Medicine, Witten/Herdecke University, 58448 Witten, Germany; frank.thevenod@uni-wh.de

**Keywords:** cadmium, embryonic kidneys, HIF-1, intrauterine exposure

## Abstract

During embryonic development, some hypoxia occurs due to incipient vascularization. Under hypoxic conditions, gene expression is mainly controlled by hypoxia-inducible factor 1 (HIF-1). The activity of this transcription factor can be altered by the exposure to a variety of compounds; among them is cadmium (Cd), a nephrotoxic heavy metal capable of crossing the placenta and reaching fetal kidneys. The goal of the study was to determine Cd effects on HIF-1 on embryonic kidneys. Pregnant Wistar rats were exposed to a mist of isotonic saline solution or CdCl_2_ (D_Del_ = 1.48 mg Cd/kg/day), from gestational day (GD) 8 to 20. Embryonic kidneys were obtained on GD 21 for RNA and protein extraction. Results show that Cd exposure had no effect on HIF-1α and prolyl hydroxylase 2 protein levels, but it reduced HIF-1 DNA-binding ability, which was confirmed by a decrease in *vascular endothelial growth factor* (*VEGF*) mRNA levels. In contrast, the protein levels of VEGF were not changed, which suggests the activation of additional regulatory mechanisms of VEGF protein expression to ensure proper kidney development. In conclusion, Cd exposure decreases HIF-1-binding activity, posing a risk on renal fetal development.

## 1. Introduction

Hypoxia plays an important role in various processes during fetal development, like placentation, angiogenesis, and hematopoiesis [1]. Renal development is also driven by low partial oxygen pressure, which in the rat initiates after the implantation and the apparition of the primitive streak, around gestational day (GD) 8 or 9 [2]. Nephrogenesis and the growth and development of renal vasculature occur simultaneously, which causes a disparity of oxygen demand and supply due to a low degree of vascularization. This generates local low oxygen tension in early developmental stages, so it is believed that hypoxia somehow regulates tissue maturation during these developmental stages [3]. Under hypoxic conditions, gene expression is primarily controlled by the hypoxia-inducible factor 1 (HIF-1). HIF-1 is a transcription factor composed by two subunits: 1α and 1β. Subunit 1β (also known as aryl hydrocarbon receptor nuclear translocator (ARNT)) is stable at any oxygen concentration, while subunit 1α is almost undetectable at normal oxygen concentrations because it is committed to proteosomal degradation [4]. Proteosomal degradation of HIF-1α is modulated by the prolyl hydroxylases 1, 2, and 3 (PHD1, PHD2, and PHD3, respectively), which hydroxylate proline residues 402 and 564 [5]. Hydroxylated subunit 1α is then recognized by the von Hippel–Lindau protein (pVHL), one of the components of the E3 ubiquitin ligase complex. This promotes its polyubiquitination and later destruction by 26s proteasome [5]. In contrast, under hypoxia HIF-1α is stabilized and translocates to the nucleus where it dimerizes with subunit 1β and activates target genes by binding to hypoxia-responsive elements (HREs) in the promoter region [4].

HIF-1 is responsible for the activation of over 60 genes that encode for growth factors, glucose transporters, transcription factors, erythropoietin, etc. In that manner, HIF-1 modulates oxygen consumption, cell survival, anaerobic metabolism, growth and development, and cell proliferation under hypoxic conditions [1,4].

During kidney development, HIF-1 appears to have important functions as well because it is present in many developing structures. For instance, HIF-1α protein is present in the nuclei of epithelial cells in ureteric buds in the medulla and cortex of human kidneys at gestational week 24. Moreover, it is found in epithelial cells of branching ampullae and S-shaped bodies in the nephrogenic zone. In rats and mice, HIF-1α is found predominantly in collecting ducts in the medulla, but rarely in the cortex [3,5]. *Vascular endothelial growth factor* (*VEGF*) is one main target gene of HIF-1 [3], and an essential molecule for normal renal development. For instance, the absence of the splicing isoforms VEGF_164_ and VEGF_188_ in mice has been associated with defective capillary angiogenesis, arteriogenesis, maturation of capillaries, development of glomerulosclerosis, and dilatation of proximal tubules and loops of Henle [6]. In addition, blocking VEGF in mice at postnatal day (PND) 0 caused a reduction on the number of nephrons and poor vascularity in the glomeruli [7]. Although the promoter region of *VEGF* has also consensus sites for Sp1/Sp3, AP-2, Egr-1, and STAT-3, HIF-1 seems to be a major determinant in the expression and secretion of VEGF during hypoxia [8]. These facts underline the importance of HIF-1 for proper kidney development.

Cadmium (Cd), a naturally occurring heavy metal, is a well-known nephrotoxicant that is capable of crossing the placenta to some extent and reaching the developing fetus [9,10]. Although a greater amount of Cd accumulates in the fetal liver, it can also reach fetal kidneys [10,11,12], and therefore, alter their development and cause damage.

A potential target for Cd toxicity during embryonic development is HIF-1. Several studies have shown that Cd may exert opposing effects on the mRNA expression and protein levels of HIF-1α, as well as on the ability of HIF-1 to bind to HREs in the promoter region of its target genes in several cell lines [13,14,15,16,17]. These differing effects could be attributed to the concentrations of Cd used, the time of exposure, and coexposure to a hypoxic stimulus. In animal models, those differences persist. For instance, in oysters, Cd exposure decreased *HIF-1α* and *PHD2* mRNA expression after postanoxic recovery [18], while under normoxia this metal ion only decreased *PHD2* mRNA expression [19]. In addition, in larval sheepshead minnow, cadmium reduced the hypoxia-induced mRNA expression of erythropoietin, a gene target of HIF-1 [20].

In spite of its nephrotoxic properties and its ability to cross the placenta, the effects of Cd exposure on embryonic kidneys are not well-investigated. Furthermore, the unavoidable exposure to this heavy metal through diet, pollution, and cigarette smoke [21] during the reproductive life stages emphasizes the importance of assessing the outcomes of the gestational exposure to Cd on this key regulator of embryonic development.

Therefore, the purpose of this study was to determine the effect of the intrauterine exposure of Wistar rats to Cd on HIF-1, its main regulator of protein stability, PHD2, and on its target gene, VEGF, in embryonic kidneys. The results show that Cd reduces HIF-1 DNA-binding ability, which was confirmed by a decrease in *VEGF* mRNA levels. However, VEGF protein levels were not altered, which suggests the activation of a compensation mechanism for appropriate kidney development.

## 2. Materials and Methods

### 2.1. Treatment of Animals and Tissue Collection

The Institutional Committee for the Care and Use of Laboratory Animals (Comité Interno para el Cuidado y uso de los Animales de Laboratorio, CICUAL) from CINVESTAV approved all animal procedures (protocol number: 041-13; approved date: 17 April 2013), and animal handling was performed in accordance with their guidelines. The samples used in this study were obtained from the animals employed in a previous study from our research group [12]. Briefly, pregnant Wistar rats were randomly divided in two groups: control (CT) and Cd, with 6 rats each. From GD 8 until GD 20, the rats from the CT group were exposed for 2 h/day to a mist of isotonic saline solution in a 10 L whole-body chamber connected to an Aeroneb nebulizer (inExpose, SCIREQ, Inc., Montreal, QC, Canada). Similarly, the rats from the Cd group were exposed by inhalation for 2 h/day to a mist of a solution of 1 mg CdCl_2_/mL (Sigma-Aldrich Co., St. Louis, MO, USA). The nebulization was controlled with the flexiWare software v.6.1. (SCIREQ, Inc., Montreal, QC, Canada, 2012) to obtain a bias flow of 3 L/min, 10% nebulization rate (0.085 mL/min), and a nebulization cycle time of 1 s. The dose concentration of the aerosol (C_Dose_) and the delivered dose (D_Del_) achieved under these conditions were 17.43 mg Cd^2+^/m^3^ and 1.48 mg Cd^2+^/kg/day, respectively. Cd exposure was applied through inhalation because it is an environmentally relevant route considering the permanent human exposure to this metal ion through air pollution and cigarette smoke [21]. Cadmium C_Dose_, however, is high compared with the Cd concentration found in the environment [21]. This decision was made taking into consideration two aspects that could affect cadmium absorption: (1) the exposure period was rather short (13 days) in contrast to human exposure, and (2) aerosol particles’ mass median aerodynamic diameter (MMAD) was larger than previous studies (2.5–3 µm) [22,23,24].

On GD 21, the dams were anesthetized with isoflurane (Sofloran Vet; PISA Farmacéutica, Hidalgo, Mexico) and the fetuses were obtained by caesarean section. The fetuses were kept in ice-cold isotonic saline solution until they were weighed and their kidneys obtained. Both kidneys of two different fetuses from each litter were homogenized independently in Trizol Reagent (Life Technologies, Carlsbad, CA, USA) the same day of extraction and homogenates were kept at −20 °C overnight until RNA extraction on the next morning.

The kidneys of the remaining fetuses were snap-frozen in liquid nitrogen and kept at −70 °C until protein extraction.

### 2.2. HIF-1 DNA-Binding Assay

A Procarta^®^ Transcription Factor Plex Kit (Affymetrix, Inc., Santa Clara, CA, USA) was used to assess the activation of HIF-1. This assay is based on the Luminex^®^ xMAP^®^ Technology (Luminex Corp., Austin, TX, USA) and it is designed to measure DNA binding of transcription factors in nuclear extracts.

A pool of fetal kidneys from each litter was used to obtain nuclear extracts according to manufacturer’s instructions. In addition, 96-well plates were prepared as indicated in the user’s manual. Individual wells were loaded with 2 μg of nuclear extracts and each sample was run in duplicate. The plate was read in a Bio-Plex^®^ System (Bio-Rad Laboratories, Inc., Hercules, CA, USA).

### 2.3. Quantitative Reverse Transcription Polymerase Chain Reaction (RT-qPCR)

As stated in Section 2.1, both kidneys from 2 fetuses from each litter were homogenized in Trizol Reagent (Life Technologies, Carlsbad, CA, USA). Homogenates were kept at −20 °C for the night, and on the next morning RNA extraction was performed according to manufacturer’s indications. RNA integrity was assessed in 1.5% agarose gels electrophoresed at 90 V for 1 h and visualized with UV light. RNA samples were stored at −70 °C until use. cDNA was synthesized from 3 μg of RNA using the ImProm-II Reverse Transcription System Kit (Promega, Madison, WI, USA) according to the manufacturer’s instructions and stored at −20 °C until its use. Purity and concentration of RNA and cDNA were evaluated using a NanoDrop 2000 (Thermo Fisher Scientific, Waltham, MA, USA).

Quantitative PCR was performed using the StepOne Real-Time PCR System (Applied Biosystems, Foster City, CA, USA) in 10 μL reactions containing: 5 μL of 2× mastermix with SYBR Green (Maxima SYBR Green/ROX qPCR Master Mix kit; Fermentas, Waltham, MA, USA), 0.1 μL of each primer solution (100 μM), 2.5 μg of cDNA and nuclease-free water to total 10 μL. Primer sequences used to assess *VEGF*, *PHD2*, HIF-1α, and *TATA-Binding Protein* (*TBP*) expression are as follows: *VEGF* 5′-TTACTGCTGTACCTCCAC-3′ (sense), 5′-ACAGGACGGCTTGAAGATA-3′ (anti-sense); *PHD2* 5′-CCATGGTCGCCTGTTACCC-3′ (sense), 5′-CGTACCTTGTGGCGTATGCAG-3′ (antisense); *HIF*-1α 5′-CCTACTATGTCGCTTTCTTGG-3′ (sense), 5′-TGTATGGGAGCATTAACTTCAC-3′ (antisense); *TBP* 5′-CACCGTGAATCTTGGCTGTAAAC-3′ (sense), 5′-CGCAGTTGTTCGTGGCTCTC-3′ (antisense). All primer sequences are published elsewhere [25,26,27,28]. Amplification was achieved according to the following cycling protocol: enzyme activation for 10 min at 95 °C, followed by 40 cycles of 15 s at 95 °C, 30 s at 60 °C, and 30 s at 72 °C. In order to assess the product specificity, a melting-curve analysis was performed.

Samples were normalized against TBP. The changes in expression relative to the CT group were calculated with the 2^−ΔΔCT^ method. All samples were amplified in duplicate. The results are presented as mean fold changes of mRNA expression levels compared with controls, which were set to 1.0.

### 2.4. VEGF Enzyme-Linked Immunosorbent Assay (ELISA)

Protein levels of VEGF were quantified in total protein extracts obtained from a pool of fetal kidneys from each litter. A commercial ELISA kit was used (RAB0512, Sigma-Aldrich Co., St. Louis, MO, USA). Reconstitution and dilution of reagents, and plate preparation were performed according to the manufacturer’s instructions. Twenty micrograms of total protein were used for each sample. All samples were run in duplicate. The color intensity of the samples was measured at 450 nm on a microplate reader (Infinite^®^ F200, TECAN Group Ltd., Männedorf, Zürich, Switzerland). The concentration of VEGF is expressed as pg/mg protein.

### 2.5. Western Blot

Aliquots of the protein extracts used for the quantification of VEGF were used to assess the protein levels of PHD2 and HIF-1α.

Protein samples (30 μg/lane) were loaded onto 12% and 10% SDS-PAGE polyacrylamide gels for PHD2 and HIF-1α detection, respectively, and run at 0.04 A for 2 h. Proteins were transferred onto 0.45 μm pore-sized nitrocellulose membranes (Bio-Rad Laboratories, Hercules, CA, USA) for 2 h at 0.4 A for PHD2, and at 0.08 A for 16 h for HIF-1α. For PHD2, the membranes were blocked for 1 h at room temperature with 5% low-fat dry milk dissolved in 0.1% PBS-Tween 20, and for HIF-1α, membranes were blocked for 1.5 h. Membranes were incubated overnight at 4 °C with rabbit primary anti-PHD2 (4835, Cell Signaling Technology, Inc., Danvers, MA, USA; diluted 1:1000) or mouse primary anti-HIF-1α antibodies (NB-100-105, Novus Biologicals, Littleton, CO, USA; diluted 1:500) in 0.1% PBS-Tween 20. Blots were washed and incubated for 1 h at room temperature with goat anti-rabbit secondary antibody (sc-2004, Santa Cruz Biotechnology, Inc., Dallas, TX, USA; diluted 1:10,000 in 0.1% PBS-tween 20) or 1.5 h with the goat antimouse secondary antibody (sc-2005, Santa Cruz Biotechnology, Inc., Dallas, TX, USA; diluted 1:10,000 in 0.1% PBS-Tween 20). After washing, proteins were detected by chemiluminescence (LuminataTM Forte, Millipore Corp., Burlington, MA, USA) using the LI-COR C-DiGit scanner (LI-COR, Inc., Lincoln, NE, USA).

Densitometric analysis was performed using the Image Studio Lite Software v.5.0.21 (LI-COR, Inc., Lincoln, NE, USA) using β-actin as a loading control.

### 2.6. Statisticals

Statistical analyses were performed with the GraphPad Prism software version 7.0 for Windows (GraphPad software, La Jolla, CA, USA). Means ± standard error of the mean (SEM) are shown. Student’s *t*-test (unpaired, two-tailed) or Mann–Whitney U-test (unpaired, two-tailed) were performed for parametric and nonparametric data, respectively. *p* ≤ 0.05 was considered statistically significant.

## 3. Results

### 3.1. Effect of Intrauterine Cadmium Exposure on DNA-Binding Ability of HIF-1 in Fetal Kidneys

Because HIF-1 is a transcription factor, its ability to bind specific sequences in the DNA is essential for gene regulation. In this study, the activity of HIF-1 was evaluated with a multiplex assay in which the samples showed higher fluorescence intensity when HIF-1 binding increased.

**Figure 1:**
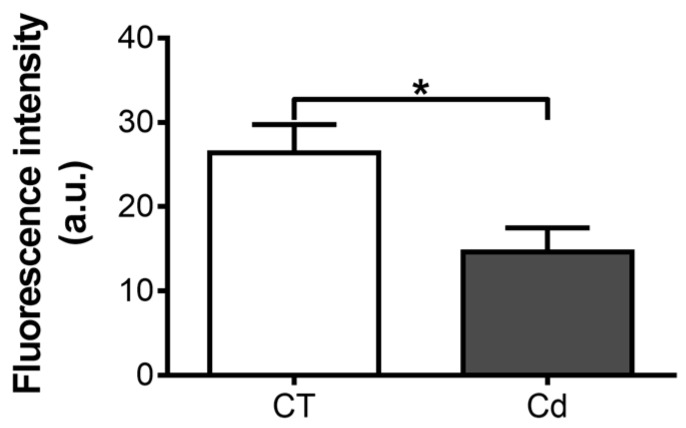
Hypoxia-inducible factor 1 (HIF-1) DNA-binding ability in nuclear extracts of kidneys of control and cadmium-exposed fetuses. Two micrograms of nuclear extracts were used per sample. Each sample was run in duplicate. The bars represent means ± SEM, *n* = 5. * *p* = 0.0307, Student’s *t*-test.

Cadmium exposure during gestation significantly reduced the ability of HIF-1 to bind DNA in fetal kidneys by 44 ± 11% (Figure 1).

### 3.2. Effect of Cadmium on the mRNA Expression of VEGF-A, PHD2, and HIF-1α in Fetal Kidneys

**Figure 2:**
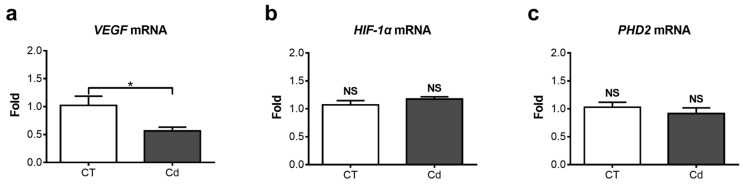
mRNA expression of (**a**) vascular endothelial growth factor (*VEGF*) (* *p* = 0.0266, Mann–Whitney U-test), (**b**) *HIF-1α* (*p* = 0.1379, Mann–Whitney U-test), and (**c**) *PHD2* (*p* = 0.4083, Student’s *t*-test) in kidneys of fetuses from control (CT) and cadmium (Cd) groups. Data were normalized with *TATA-Binding Protein* (*TBP*) expression. Each sample was run in duplicate. The fold changes compared to the CT group are plotted. Means ± SEM are shown, *n* = 9. NS, not statistically significant.

The mRNA expression of *VEGF* was assessed since it is an important target gene of HIF-1, which has been associated with normal kidney development. Cadmium exposure significantly decreased the expression of *VEGF* by 44 ± 7% in fetal kidneys compared with the CT group (Figure 2a). However, Cd exposure did not alter the expression of *HIF-1α* or of *PHD2*, which promotes HIF-1α degradation (Figure 2b,c).

### 3.3. Cadmium-Induced Changes of VEGF, PHD2, and HIF-1α Protein in Fetal Kidneys

**Figure 3:**
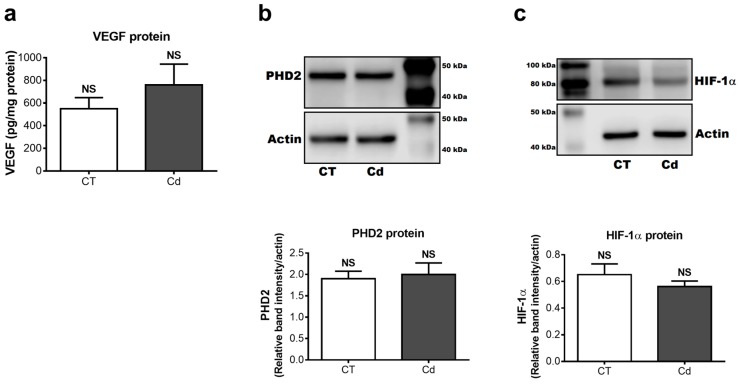
Protein levels of (**a**) VEGF (*p* = 0.3389, Student’s *t*-test), (**b**) PHD2 (*p* = 0.7702, Student’s *t*-test), and (**c**) HIF-1α (*p* = 0.3476, Student’s *t*-test) in renal tissue from control and Cd-exposed fetuses. (**b**,**c**) show representative blots of one of six samples from each group, and densitometry. Band intensities were normalized to actin level. VEGF concentration was normalized to protein content. Each sample was run in duplicate. Means ± SEM are shown, *n* = 6. NS, not statistically significant.

To complement gene expression, protein levels of VEGF, PHD2, and HIF-1α were also assessed. In contrast to expectation, VEGF protein levels in the kidneys of Cd-exposed fetuses (759.2 ± 184.7 pg/mg total protein) did not differ from those of the control group (548.8 ± 98.9 pg/mg total protein) (Figure 3a). Similarly, the densitometric analysis showed no difference in the relative levels of PHD2 and HIF-1α of the CT and Cd groups (Figure 3b,c), which was in accordance with the findings of their gene expression.

## 4. Discussion

The long-term exposure, even at low doses, of pregnant women to heavy metals capable of accumulating in the body can generate irreversible outcomes in fetal growth and development [29]. Cd intoxication is a common danger for all organisms because of its continuing presence in polluted air and tobacco smoke that, when inhaled by pregnant women, poses a serious threat to the woman and, particularly, the developing fetus due to lack of (or minimal presence of) mechanisms of protection [29].

Because of this, it is important to characterize the effects of gestational exposure of Cd metal ion and find its possible targets. As stated in the introduction section, one of them is HIF-1, one of the main transcription factors that control the expression of several genes that are necessary for cell survival and proliferation, and glucose metabolism [4] under hypoxic conditions, such as during embryonic development.

The expression of its subunit 1α, as well as its activity, can be modified by the exposure to several metal ions, like cobalt, nickel [14,30,31], and Cd. The aforementioned metal ions increase the mRNA and/or protein levels of HIF-1α, as well as the ability of HIF-1 to bind to its HREs. For Cd, however, conflicting results have been obtained. Several studies in cell lines, as well as animal models, have shown that Cd increases HIF-1α protein levels and HIF-1 activity, as reflected by a rise in VEGF transcription [16,32]. Nevertheless, in HEK293 and Hep3B cells under a hypoxic stimulus, Cd decreased the ability of HIF-1 to bind DNA [13,15,17] because of increased proteasomal degradation of subunit 1α [13].

In the light of these observations, the goal of the study was to assess the effects of Cd exposure on HIF-1 in kidney tissue during a highly vulnerable stage, namely, gestational development.

The effectiveness of the parameters of exposure (described in Section 2.1) was evaluated by measuring the content of this metal ion in dam and fetal organs such as lungs, liver, kidneys, placentae, and fetal kidneys. The cadmium burden on those organs was significantly greater than in the control group [12], demonstrating an effective absorption and distribution of Cd in the dam, and, more importantly, showing that this transition metal ion crosses the placenta and reaches developing kidneys. Moreover, dam organs did not show any changes in relative weight, and mothers did not present signs of overt toxicity [12], suggesting that all changes observed in fetal kidneys were direct effects of Cd.

This study presents evidence that gestational exposure to Cd decreases HIF-1 DNA-binding ability in embryonic kidneys without altering the mRNA or protein levels of its subunit 1α, which is in accordance with the lack of change in PHD2 mRNA and protein levels.

So far, the mechanism by which Cd could be exerting its inhibitory effect on HIF-1 activity without modifying HIF-1α protein levels has not been elucidated. However, Kubis et al. [33] reported a cytoplasmic accumulation of the subunit 1α in a skeletal muscle primary culture from New Zealand rabbits exposed to Cd and subjected to hypo-(3% O_2_) and hyperoxic (42% O_2_) conditions. This caused a decrease of the translocation of HIF-1α to the nucleus and lower mRNA levels of *glyceraldehyde-3-phosphate dehydrogenase* (*GAPDH*), one of HIF-1’s target genes. The authors attributed the lower nuclear import and, therefore, DNA-binding ability to a higher association of subunit 1α to heat shock protein 90 (Hsp90). Hsp90 is a chaperone that prevents the aggregation of un- or misfolded proteins produced under stress situations [34]. Additionally, it takes part in von Hippel–Lindau-independent regulation of HIF-1. Under normoxic conditions, Hsp90 binds to the basic helix-loop-helix-Per-ARNT-Sim (bHLHL-PAS) domain found in subunit 1α, which stabilizes the protein and prevents it from being degraded, but keeps the subunit in an inactive state. Under hypoxic conditions, the binding loses its strength, allowing the nuclear translocation of the subunit and further binding to its HREs [34,35,36].

In this study, we assessed neither Hsp90 levels nor its binding to subunit 1α in embryonic kidneys, but previous reports indicate that basal levels of this protein in newborn kidneys from both humans and rats (PND 1) is higher than in adult kidneys [37,38]. In fetal kidneys, Hsp90 is expressed in the parietal epithelium of Bowman’s capsule, podocytes, blastema, S-shaped bodies, proximal convoluted and straight tubules, as well as collecting ducts [37,38]. Furthermore, several studies associated Cd exposure with increased Hsp90 levels. For instance, Leghorn chick embryos exposed *in ovo* to Cd for 24 h had higher protein levels of Hsp24, Hsp70, and Hsp90 than those in the control group [39]. Similar increases were found in renal tissue from rat [40], duck [41], carp [42], and the proximal tubule cell line, NRK-52E exposed to Cd [43]. More importantly, a study showed that increasing doses of Cd (0.5–4 mg Cd^2+^/kg, i.p.) resulted in a dose-dependent increase in the level of association between Hsp90 and one of its substrates, the glucocorticoid receptor, in liver cytosolic extracts [44]. Putting these findings together, it is plausible that Cd increases the degree of association between subunit 1α and Hsp90 by a process (still to be identified) without altering the protein levels of 1α subunit, thus leading to a cytoplasmic accumulation of the subunit and lower DNA-binding ability of HIF-1. This hypothesis needs to be tested in our model of exposure by determining HIF-1α nuclear and cytoplasmic levels, as well as its association to HSP90.

Another possible explanation relies on the fact that HIF-1 requires copper to bind its HREs because it promotes complexation of HIF-1α with its cofactor p300 through inhibition of the factor inhibiting HIF-1 (FIH-1) [45]. It has been shown that Cd exposure decreases the maternal transfer of micronutrients such as iron, zinc, and copper, thus altering their burden in several organs [46,47]. This raises the possibility that HIF-1 activity may be decreased by a lower copper content in fetal kidneys, as previously reported [48]. This speculation requires, of course, further experimental confirmation since contrasting effects have been found on this matter [46,48,49], probably due to dose, route, length, and period of exposure.

Consistent with a lower HIF-1 DNA-binding ability, *VEGF* mRNA levels were significantly reduced by Cd exposure, which confirms a reduced activity of this transcription factor. This Cd-induced effect was previously reported by Gheorghescu et al. [50]. The authors showed that the exposure of chick embryos from the Ross strain to a 50 µM Cd acetate solution decreased *VEGF-A* mRNA expression in extraembryonic membranes 1 h post-treatment [50]. In contrast to the mRNA levels, VEGF-A protein expression was not modified by Cd exposure. This difference could be due to pleiotropic regulation of this growth factor. At the translational level, VEGF is regulated by internal ribosome entry sites, upstream open reading frames, alternative initiation codons, micro-RNAs, riboswitches, and RNA G-quadruplex structures [51]. This is understandable considering the importance of this molecule for angiogenesis and vasculogenesis and, therefore, fetal development. Thus, it is possible that the rate of protein translation was increased or post-translational modifications of VEGF affected by any of those mechanisms to compensate the lowered mRNA levels induced by Cd. Nevertheless, further corroboration is needed.

It is noteworthy that the present study serves as a first approach to try to elucidate molecular targets of cadmium in developing organisms; hence, it is important to do further assessments that will help to understand more clearly the detrimental effects (not only renal) of this transition metal after gestational exposure. This should include the evaluation of the relative expression of other HIF-1 target genes, as well as their protein levels and the degree of their functionality, if possible. The expression of miRNAs should also be evaluated, since they participate in the post-transcriptional regulation of gene expression and might have an important role during renal (and embryonic overall) development. In addition, an alternative approach could include the use of cellular models from embryonic origin to confirm these findings and evaluate a possible mechanism by which cadmium is impairing HIF-1 DNA-binding, as well as other molecular targets.

## 5. Conclusions

The results of this study show that Cd in utero exposure impairs HIF-1 DNA-binding activity in developing kidneys, by a mechanism that is independent of HIF-1α protein levels and needs to be identified. This observation is in accordance with previous reports. In addition, reduced transcriptional activity of HIF-1 was confirmed by lower *VEGF* mRNA levels although protein levels remained unchanged. This finding suggests the existence of alternative compensatory mechanisms to maintain adequate protein levels of this key molecule and thus ensure proper fetal development. Nevertheless, it is important to study further possible outcomes of decreased HIF-1 activity, as well as other mechanisms of Cd toxicity targeting HIF-1, because embryonic development is highly vulnerable, and any alteration during this stage can lead to developmental defects later in life.
